# Induction of lipid A modification genes in Pseudomonas aeruginosa cells tolerant to a commercially available contact lens disinfection solution

**DOI:** 10.1099/jmm.0.002073

**Published:** 2025-10-03

**Authors:** Yasmin Hilliam, Stuart D. Armstrong, R.F. Langendonk, Stephen Kaye, Craig Winstanley

**Affiliations:** 1 Institute of Infection,Veterinary & Ecological Sciences, University of Liverpool, Liverpool, UK; 2 Institute of Life Course & Medical Sciences, University of Liverpool, Liverpool, UK

**Keywords:** contact lens solutions, drug tolerance, keratitis, proteome, *Pseudomonas* infections, transcriptome

## Abstract

**Introduction.** *Pseudomonas aeruginosa* has a high propensity to develop drug resistance and is a leading cause of bacterial keratitis, particularly amongst contact lens (CL) wearers. Although CL wearers are advised to regularly disinfect their CLs with fresh multi-purpose disinfection solutions, adherence is often poor. This raises the possibility that *P. aeruginosa* may develop resistance to multi-purpose disinfection solutions when exposed to sub-inhibitory concentrations of disinfection solutions.

**Aim.** We therefore investigated the survivability of a CL-associated keratitis isolate of *P. aeruginosa* (PA76203) in a commercially available multi-purpose disinfection solution (Opti-Free RepleniSH) when pre-treated with a sub-inhibitory concentration of solution.

**Methodology.** Survival of PA76203 in 100% (v/v) disinfectant solution was evaluated for up to 6 h with both untreated control cultures and cultures pre-treated with 30% (v/v) solution, using bacterial colony count data. Transcriptomic and proteomic analysis of the model strain *P. aeruginosa* PAO1 was conducted to evaluate genes and proteins associated with growth in 30% (v/v) solution.

**Results.** Untreated PA76203 was undetectable in the disinfection solution after 10 min incubation, whereas pretreated PA76203 was detectable at 6 h (*P*<0.05), indicating the acquisition of a tolerance phenotype. Transcriptomic and proteomic data from PAO1 treated with a sub-inhibitory concentration of disinfectant revealed 85 significantly differentially expressed genes and 342 differentially abundant proteins, respectively. Genes and proteins involved in LPS lipid A modifications (including *arnA*, which encodes the first functional enzyme in a lipid A modification operon) were shown to be upregulated in the pre-treated condition compared to the untreated control. The tolerance phenotype was not maintained in a strain of *P. aeruginosa* with a non-functional *arnA* gene.

**Conclusion.** Exposure of *P. aeruginosa* to sub-inhibitory concentrations of disinfection solution enhances tolerance to previously lethal concentrations of solution and is positively associated with upregulation of genes involved in LPS lipid A modifications.

## Graphical abstract

*Pseudomonas aeruginosa* survival in a commercially available contact lens disinfection solution is increased by exposure to sub-inhibitory concentrations of the solution via induction of lipid A modification pathways. Created in BioRender. Hilliam, Y. (2025) https://BioRender.com/w7jkvrj.

**Impact Statement**

*Pseudomonas aeruginosa* is a leading cause of eye infections, particularly in contact lens (CL) users. Compliance with using CL disinfection solutions is variable, exposing *P. aeruginosa* to non-lethal concentrations of such solution. We show that pre-treatment of *P. aeruginosa* with 30% concentrations of contact lens disinfection solution enhances tolerance to previously lethal 100% concentrations of solution, likely due to upregulation of genes associated with LPS lipid A modifications.

## Data Summary

The authors confirm that all supporting data, code and protocols have been provided within the article or through supplementary data files. The individual accession numbers are SRR32923714, SRR32923713, SRR32923718, SRR32923717, SRR32923716 and SRR32923715.

## Introduction

Myopia (nearsightedness) is increasing worldwide due to changes in lifestyle [1,5]. Global increases in myopia and industrialization have led to corresponding increases in the use of vision-correcting contact lenses (CLs) [6]. Consumer compliance with ophthalmologist-recommended hygiene regimes is key in reducing the risk of serious complications associated with CL use [7]. Previously, many CL users cleaned their lenses with hydrogen peroxide-based solutions which posed little risk for development of resistance or tolerance, but in recent decades, multi-purpose CL disinfectant solutions have become increasingly popular. This shift away from disinfection solutions that require neutralization (hydrogen peroxide-based) or manual lens cleaning is driven by an assumption that consumers are more likely to adhere to a simpler routine. Even amongst CL wearers that self-report good compliance with their recommended hygiene routines, actual compliance remains poor [8].

Many of these multi-purpose solutions contain disinfectants that do not require neutralization or manual cleaning of lenses to remove particulates before the CL can be used. Quaternary ammonium compounds (QACs) are a group of important disinfectants and are amongst the most commonly used active ingredients in CL disinfection solutions. QACs are used for disinfection in the home, in industry and in healthcare settings. They are believed to act to disrupt the bacterial cell membrane, although little is known currently about their exact mode of action [9,10]. As with antibiotics, there are fears that overuse of disinfectants within the human environment could drive the development of resistance, which presents a threat to human health in both home and healthcare environments.

*Pseudomonas aeruginosa* is an opportunistic bacterial pathogen that is a leading cause of bacterial keratitis [11,12]. It boasts a large, plastic genome that confers intrinsic resistance to many conventional antibiotics and allows for the development of resistance to others [13]. During infection, *P. aeruginosa* can form biofilms which further contribute to intrinsic antimicrobial resistance, including protecting the bacteria from host pathogen responses [13]. *P. aeruginosa* is commonly found in potable water sources associated with humans and so poses a significant contamination risk for CL users.

Here, we provide evidence for the development of phenotypic tolerance to a commercially available multi-purpose disinfection solution in a clinical isolate of *P. aeruginosa* after exposure to a suboptimal concentration of a CL disinfectant solution. Alterations to the transcription and translation of genes associated with polymyxin B resistance were shown to be responsible for the increased survival of *P. aeruginosa* in a disinfection solution [14]. These data shed light on the potential killing mechanisms and bacterial evasion of QACs as disinfectants and raise concerns about the subsequent effect on the development of antibiotic resistance in clinical strains of *P. aeruginosa*.

## Methods

### Bacterial strains

#### *P. aeruginosa* 76203

Isolate 76203 was acquired as part of the UK Microbiology Ophthalmic Group collection of keratitis isolates from eye care centres in the UK [15]. 76203 was collected from a patient who presented with a CL-associated keratitis infection in 2007. The cultured isolate was stored at −80 °C in 5% (v/v) glycerol in Luria broth (LB) for future use.

#### PAO1 and PW7025 transposon mutant

Strain PAO1 (Washington) and transposon mutant PW7025 were both acquired from the *P. aeruginosa* mutant library held by the Manoil Laboratory at the University of Washington, USA. PAO1 (Washington) is used as the background strain for the transposon mutants listed in the library [16,17]. Strain PW7025 (genotype arnA-E01:: ISphoA/hah) has a transposon insertion in *arnA* (locus PA3554) which is predicted to render it non-functional.

### Determination of sub-inhibitory concentration of CL disinfection solution

MIC of Opti-Free RepleniSH against *P. aeruginosa* PAO1 was determined by OD growth curve assay. A 1 : 2 serial dilution of Opti-Free RepleniSH in LB was made in a flat-bottom 96-well plate (50%–0.05%) with positive, negative and agent controls. OD was measured at 625 nm every 30 min for 16 h. Only the 50% concentration showed substantial inhibition of growth (Fig. S1), and so a concentration of 30% (v/v) was determined to be suitable as a sub-inhibitory concentration. There was no difference in the growth of PAO1 and 76203 in 30% (v/v) Opti-Free RepleniSH and LB (Fig. S2).

### Bacterial survival time assay in CL disinfection solution

Bacterial isolates were grown for 16 h in either untreated or pre-treated conditions. The untreated condition constituted growth in 5 ml 30% (v/v) sterile distilled water in LB at 37 °C on a platform shaker at 180 r.p.m. The pre-treated condition constituted growth in 5 ml 30% (v/v) Opti-Free RepleniSH in LB at 37 °C on a platform shaker at 180 r.p.m. Following growth to the late stationary phase, 1 ml of culture was centrifuged to form a pellet. The supernatant was removed, and the pellet was resuspended in sterile PBS. Washed cells were adjusted to 0.1±0.005 OD_600_. Per International Standards Organization guidelines [18], washed cells were inoculated into 100% (v/v) Opti-Free RepleniSH at a concentration of 1×10^6^ c.f.u. ml^−1^ in 10 ml. At each time point, the inoculated disinfection solution was mixed by inversion, and 100 µl was removed and added to 900 µl Dey–Engley Neutralizing Broth (MilliporeSigma, MA, USA) in order to halt any further killing by the disinfection solution. Cells were serially diluted and plated on tryptic soy agar (Neogen, MI, USA) for colony counting. Count plates were inoculated at 37 °C overnight.

### Preparation of bacterial cultures for transcriptome and proteome analysis

A single bacterial colony was picked and suspended in 5 ml LB and incubated overnight at 37 °C with shaking at 180 r.p.m. Overnight cultures were adjusted to 0.1±0.05 OD_600_ with fresh LB. Adjusted culture was used to inoculate 5 ml of untreated control media or pre-treated media as described above at a concentration of 1 : 100. Cultures were grown at 37 °C with shaking at 180 r.p.m. until the late stationary phase was reached (16 h) to recapitulate an overnight soak in CL disinfecting solution. Cultures for proteomic analysis were grown in four replicates (i.e. 20 ml total volume) in order to be pooled to provide sufficient total protein.

### RNA purification and sequencing

Total RNA was extracted using the Zymo Direct-zol RNA Miniprep Plus kit (Zymo Research, CA, USA). Two hundred microlitres of liquid culture were pipetted into a DNase/RNase-free microcentrifuge tube and centrifuged at 13,000 r.p.m. for 1 min. The supernatant was poured off, and the cell pellet was resuspended in 600 µl TRI Reagent (Sigma-Aldrich, MO, USA) and centrifuged at 13,000 r.p.m. for 30 s. The supernatant was then transferred to a fresh DNase/RNase-free microcentrifuge tube, and one volume of 100% (v/v) ethanol was added and mixed thoroughly before being transferred to a Zymo-Spin IIICG column in a 2 ml collection tube. RNA extraction was performed as per the manufacturer’s instructions and was eluted in 100 µl DNase/RNase-free water. Additionally, eluted RNA was treated using the TURBO DNA-free kit (Invitrogen, MA, USA) following the manufacturer’s instructions to ensure complete removal of DNA and enzymes in the final eluate. The extracted RNA was further purified using Agencourt RNAClean XP magnetic beads (Beckman Coulter, CA, USA). RNA was bound to the beads using Agencourt RNAClean XP solution and held in place by a magnetic rack, whilst the beads were washed with ethanol. After three washes, beads were allowed to air dry to ensure complete evaporation of ethanol before purified RNA was eluted in 40 µl DNase/RNAse-free water.

RNA library preparation and sequencing was carried out at the Centre for Genomic Research at the University of Liverpool, Liverpool, UK. rRNA depletion was performed on 0.5 µg total RNA from each sample using a Bacteria Ribo-Zero Magnetic kit (Illumina, CA, USA). Depletion was checked visually on a Bioanalyzer RNA Pico chip (Agilent Technologies, California, USA). RNAseq libraries were prepared from enriched RNA samples using the NEBNext Ultra 11 Directional RNA Library Prep kit for Illumina (New England Biolabs, MA, USA). Samples were fragmented by heating at 94 °C and used to make double-stranded cDNA. Adapters were ligated to the ends, and the constructs were amplified with index primers for nine cycles. Libraries were then purified using AMPure XP beads (Beckman Coulter, CA, USA). Each library was quantified using Qubit 3.0 fluorometer (Invitrogen, MA, USA), and the size distribution was assessed using the Agilent 2100 Bioanalyzer (Agilent Technologies, CA, USA). Clustering of the cDNA templates was performed using a HiSeq 3000/4000 Paired-End Cluster kit with a cBot Cluster Generation System (Illumina, CA, USA), according to the manufacturer’s instructions. Following cluster generation, libraries were sequenced on a HiSeq 4000 (Illumina, CA, USA) to generate 2×150 bp paired-end reads.

### Transcriptomic analysis

Initial processing quality assessment of the sequence data was performed using an in-house pipeline at the Centre for Genomic Research, Liverpool. Basecalling and de-multiplexing of indexed reads were performed by CASAVA (RRID:SCR_001802) version 1.8.2 (Illumina, CA, USA) to output samples in FASTQ format. The raw FASTQ files were trimmed to remove Illumina adapter sequences using cutadapt (RRID:SCR_011841) version 1.2.1 (1). The option ‘-O 3’ was set, so the 3′ end of any reads which matched the adapter sequence over at least 3 bp was trimmed off. The reads were further trimmed to remove low-quality bases using Sickle (RRID:SCR_006800) version 1.200 with a minimum window quality score of 20. After trimming, reads shorter than 20 bp were removed.

Trimmed reads were quality-checked using FastQC (RRID:SCR_014583) version 0.11.8 before being aligned to a *P. aeruginosa* PAO1 (RefSeq accession: GCF_000006765.1) reference genome using HISAT2 (RRID:SCR_015530) version 2.2.1 [19]. Generated alignment files were sorted, and count tables were generated using the featureCounts (RRID:SCR_012919) function of the program subread version 2.0.1 [20]. Differential expression analysis between control and pre-treated conditions was performed using DESeq2 (RRID:SCR_015687) version 1.36.0 [21]. All data were visualized using R Project for Statistical Computing (RRID:SCR_001905) version 4.2.1. Data and analysis code for this manuscript can be accessed in a repository at https://github.com/yasminhilliam/PaTolerance.

### Protein preparation and quantification

After growth, cultures were centrifuged in 15 ml centrifuge tubes at 10,000 ***g*** for 5 min, and the supernatant was removed. All pellets for each sample were resuspended in 1 ml of sterile PBS and centrifuged at 10,000 ***g*** for 1 min. The supernatant was poured off, and the cell pellet was stored at −80 °C before submission. Protein quantification and proteomic analysis were carried out as described by Bricio-Moreno *et al*. [14].

### Statistical analysis

The count data were normalized by the addition of 0.01 to all count data to allow for statistical testing at time points where count totals were zero. Data were compared between strain or treatment and per time point by ANOVA with Tukey post-hoc test, with a significance cut-off of 0.05. Tukey tables are shown in the supplemental materials (Tables S1 and S2, available in the online Supplementary Material).

## Results

### Pre-treatment with a sub-inhibitory concentration of disinfectant increases the survival of a clinical isolate in the same disinfectant

**Fig. 1.**
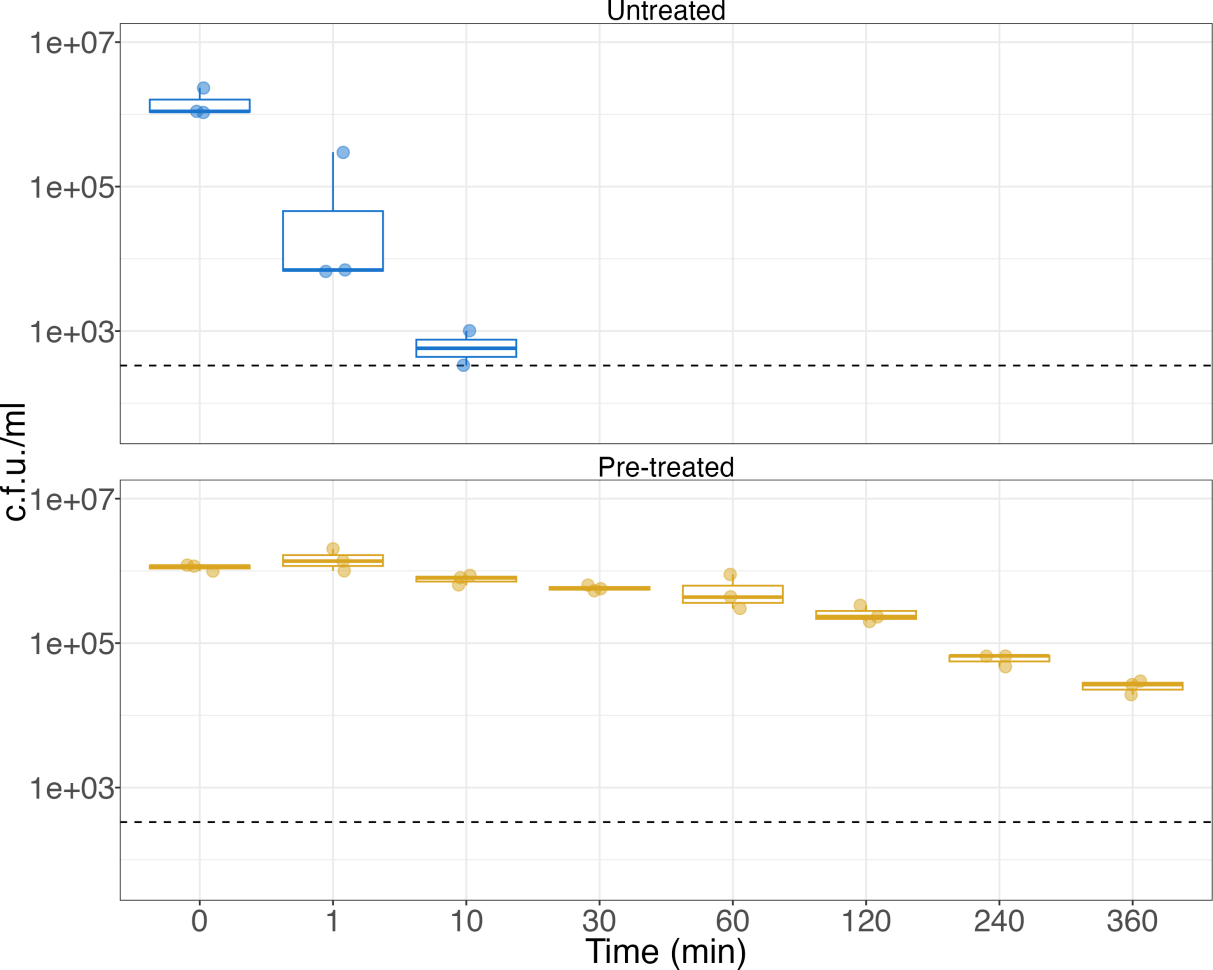
Pre-treatment with a sub-inhibitory concentration of Opti-Free RepleniSH increases survival time of a susceptible clinical isolate of *P. aeruginosa*. Colony count data at each recorded time point for *P. aeruginosa* 76203 grown in untreated (top panel; blue) and pre-treated (bottom panel; gold) conditions show that following pre-treatment *P. aeruginosa* 76203 is still detectable in high numbers after 6 h of incubation in Opti-Free RepleniSH. Survival across the time course was shown to be significantly different between pre-treated and untreated conditions (*P*<0.001) by ANOVA and Tukey post-hoc test. A horizontal dashed line indicates the limit of detection. The lower and upper hinges correspond to the first and third quartiles. The upper whisker extends from the hinge to the largest value no further than 1.5 times the interquartile range (IQR) from the hinge, and the lower whisker extends from the hinge to the smallest value at most 1.5 times the IQR of the hinge. Each point represents the mean count from a biological replicate of three technical replicates.

Untreated *P. aeruginosa* 76203 was undetectable in the disinfection solution after 10 min of incubation at room temperature (Fig. 1). However, when grown to mid-log phase in 30% (v/v) Opti-Free RepleniSH prior to inoculation, the isolate was still detectable at 1.9×10^4^ c.f.u. ml^−1^ after 6 h of incubation in full-strength Opti-Free RepleniSH. Growth across the time course was shown to be significantly different between untreated and pre-treated conditions (Table S1, ANOVA with Tukey post-hoc test, *P*<0.05).

### Genes and proteins involved in LPS restructuring to mitigate killing by antimicrobial peptides are increased in pre-treated *P. aeruginosa*

**Fig. 2.**
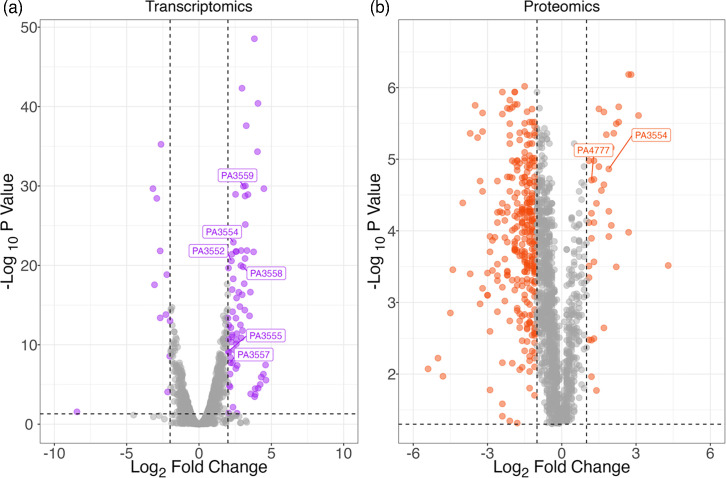
RNA sequencing and proteomic analysis revealed significantly altered gene expression and protein levels following pre-treatment with a sub-inhibitory concentration of Opti-Free RepleniSH. (a) Volcano plot representing differentially expressed genes when *P. aeruginosa* PAO1 is pre-treated with Opti-Free RepleniSH. Vertical dashed lines indicate a log_2_ fold change cut-off of < −2 and >2. A horizontal dashed line indicates a significance cut-off of adjusted *P* value<0.05. Genes of interest are annotated with their PA gene locus ID. (b) Volcano plot representing differentially abundant proteins when *P. aeruginosa* PAO1 is pre-treated with Opti-Free RepleniSH. Vertical dashed lines indicate a log_2_ fold change cut-off of < −1 and >1. A horizontal dashed line indicates a significance cut-off of adjusted *P* value<0.05. Proteins of interest are annotated with their PA gene locus ID. Abbreviations: ID, identification; PA, *P. aeruginosa*.

**Fig. 3.**
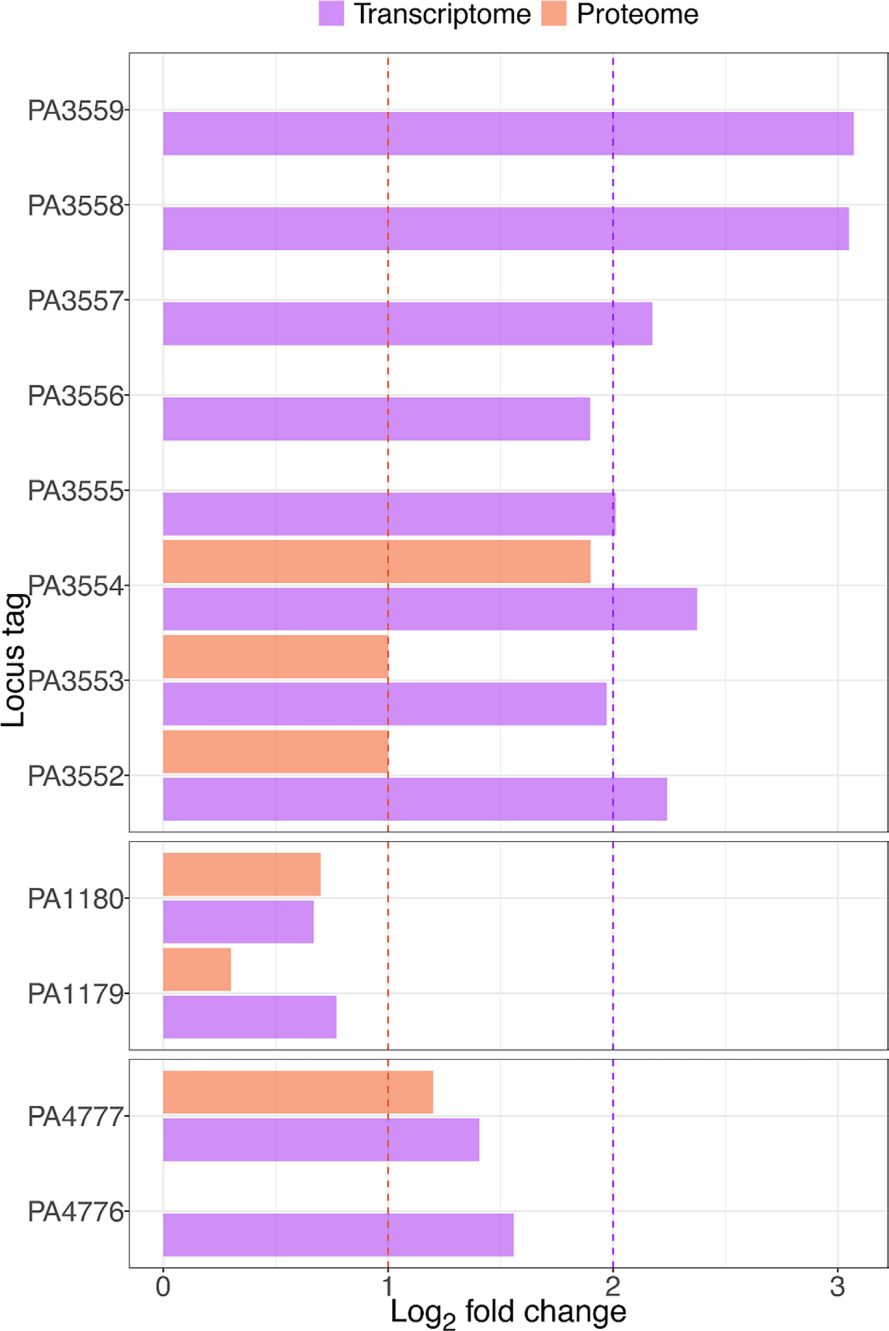
Genes involved in lipid A modification, regulators of these genes and their transcribed proteins are upregulated in PAO1 when pre-treated with Opti-Free RepleniSH. Panels are separated by functional operon, as defined by the database for prokaryotic operons [37], and log_2_ fold change data from proteomics are shown in orange, and data from transcriptomics are shown in purple. Log_2_ fold change cut-off values are indicated by dashed lines in purple (transcriptome) and orange (proteome). As expected, not all corresponding proteins are upregulated when the genes encoding them are upregulated. Loci for which upregulation was detected for both the gene expression and protein abundance are annotated with their known gene names. Abbreviation: PA, *P. aeruginosa*.

Transcriptomic data from *P. aeruginosa* PAO1 treated with a sub-inhibitory concentration of Opti-Free RepleniSH revealed 85 significantly differentially expressed genes (Fig. 2a). Proteomic data of the same treatment revealed 342 differentially abundant proteins (Fig. 2b). Genes and proteins involved in LPS lipid A modifications were shown to be upregulated in the pre-treated condition vs. the untreated control (Fig. 3).

### Pre-treatment with a sub-inhibitory concentration of disinfectant does not rescue the survival of an *arnA*-deficient transposon mutant of *P. aeruginosa*

**Fig. 4.**
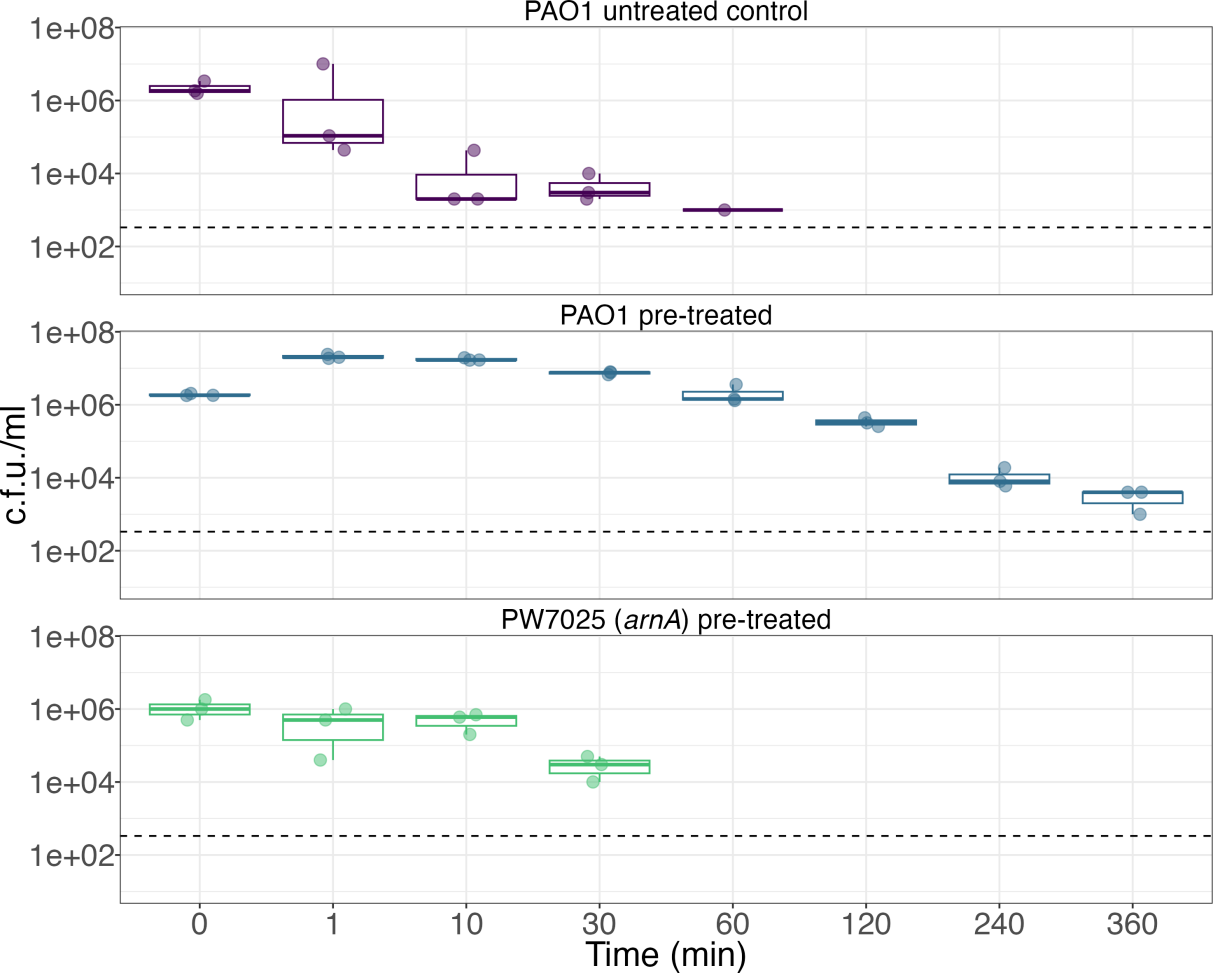
Tolerance phenotype is not maintained in a strain of *P. aeruginosa* with a non-functional *arnA* gene. Colony count data at each recorded time point for *P. aeruginosa* PAO1 grown in untreated (control; top panel, purple) and pre-treated (middle panel, teal) conditions compared to pre-treated transposon mutant PW7025 (bottom panel, green). A horizontal dashed line represents the limit of detection. Survival across the time course was shown to be significantly different between pre-treated PAO1 and PW7025 (*P*<0.001) by ANOVA and Tukey post-hoc test. The lower and upper hinges correspond to the first and third quartiles. The upper whisker extends from the hinge to the largest value no further than 1.5 times the IQR from the hinge, and the lower whisker extends from the hinge to the smallest value at most 1.5 times the IQR of the hinge. Each point represents the mean count from a biological replicate of three technical replicates. Abbreviation: PA, *P. aeruginosa*.

We assessed the importance of *arnA* on the tolerance phenotype by pre-treating PW7025 with a sub-inhibitory concentration of Opti-Free RepleniSH before inoculating into undiluted disinfecting solution. Pre-treated PW7025 survived for less time than untreated PAO1 and was not detectable after 30 min of incubation in Opti-Free RepleniSH at room temperature (Fig. 4). Growth across the time course was shown to be significantly different between pre-treated PAO1 and PW7025, and there was no difference between survival over time between untreated PAO1 and PW7025 (Table S2, ANOVA with Tukey post-hoc test, *P*>0.05).

## Discussion

One of the proteins identified as playing a potential role in the development of tolerance to Opti-Free RepleniSH in *P. aeruginosa* is ArnA (Figs 2 and 3), a bifunctional polymyxin resistance protein [22]. Expression of PA3554 (*arnA*) was shown to be upregulated in transcriptomic data, and ArnA protein abundance was shown to be higher in proteomic data when pre-treated with Opti-Free RepleniSH. The role of ArnA in the development of tolerance was investigated further through the use of a transposon mutant (Fig. 4) acquired from the Manoil Laboratory at the University of Washington, USA [16,17]; survival time in Opti-Free RepleniSH of the non-functional *arnA* transposon mutant was decreased following growth in a sub-inhibitory concentration of Opti-Free RepleniSH when compared to PAO1 grown in the same pre-treated condition. *arnA* (also known as *pmrI*) is a member of the *arnBCADTEF*-PA3559 (PA3552–PA3559) operon (referred to henceforth as the *arn* operon). The genes encoded in this operon are conserved across many pathogenic bacterial species, including *Escherichia coli*, *Yersinia pestis*, *Burkholderia cepacia* complex and all *Salmonella* [23]. Expression of this operon is regulated by the two-component regulatory system *pmrAB* [24]. The function of the *arn* operon is to modify the LPS structure of the outer membrane of Gram-negative bacterial cells. The conserved element of LPS is lipid A, and it is commonly recognized as a pathogen-associated molecular pattern in the host immune response. Therefore, one of the most common methods of immune evasion is the modification of lipid A and the structure of the LPS membrane [25]. *arnA* encodes the first pathway-specific enzyme which leads to modification of lipid A in the membrane LPS through the addition of 4-amino-4-deoxy-l-arabinose (Ara4N) to the phosphate groups in lipid A. This modification neutralizes the negative charge on the phosphate, adding a sugar with a positively charged amine. This change to the charge of the membrane reduces the binding affinity for cationic antimicrobial peptides (CAMPs) [25].

The active ingredient in Opti-Free RepleniSH is polyquaternium-1, with EDTA acting as a preservative. Whilst little is known about the precise target and mode of action of polyquaternium-1, it has been shown in previous works that polyquaternium-1 (and other QACs) predominantly targets the cytoplasmic membrane of cells [26]. In the case of *P. aeruginosa* and other Gram-negative bacteria, QACs cause damage to the outer membrane through cationic interactions with charged membrane molecules which allows for self-promoted uptake to the inner membrane of the cell where the disinfectant can act to disorganize the membrane and allow leakage of important cellular materials [26,28]. Although the main role of EDTA in Opti-Free RepleniSH is as a preservative, EDTA is also known to disrupt the LPS bilayer of Gram-negative bacteria and increase permeability of the cells and likely also plays a role in triggering lipid A modification in *P. aeruginosa* [29]. In order to define the relative contributions of polyquaternium-1 and EDTA to the observed transcriptional and proteomic effects, further experiments would be required. The work presented in this study suggests that the cationic nature of Opti-Free RepleniSH allows for the development of resistance and tolerance through adjustments to the LPS, specifically through expression of the conserved *arn* operon and alteration of lipid A molecules.

We provide compelling evidence for the role of lipid A modification mediated by the *arn* operon in the increased survival time observed in pre-treated *P. aeruginosa* 76203, and further work is required to investigate the exact mechanisms of the response. As *arnA* is the first functional enzyme in the pathway leading to the addition of Ara4N to lipid A, it is likely that any mutation giving rise to a non-functional ArnA protein would have the same deleterious effect on the development of a tolerance phenotype.

However, in order to corroborate these findings, it would be necessary to create both a knock-out mutant and a complemented mutant in order to ascertain if the tolerance phenotype is restored when the *arnA* gene is correctly transcribed. Further transposon mutant experiments could also investigate the effect of knocking out the remaining *arn* operon genes and both two-component regulators (PmrAB and PhoPQ). In work on *Salmonella enterica*, the genes in the *pmr* operon were necessary for the modification of lipid A through the addition of Ara4N, except for *pmrM* (*arnF*) [30], so it is likely that the development of loss-of-function mutants in these genes in *P. aeruginosa* would yield similar results. The *pmr* operon as described in *S. enterica* and other organisms contains seven gene members; the *arn* operon in *P. aeruginosa* has eight gene members [31,32]. The *arn* operon in *P. aeruginosa* includes PA3559 which is predicted to encode a uridine diphosphate (UDP)-glucose/guanosine diphosphate-mannose dehydrogenase (InterPro ID: IPR016040). It is likely that this enzyme plays a role in the early stages of lipid A modification, before catalysation by ArnA (i.e. by pathway-specific enzymes) occurs. The proposed pathway for the addition of Ara4N to lipid A begins with oxidation of UDP-glucose by UDP-glucose dehydrogenase to form UDP-glucuronic acid [23,25], and so it is likely that co-transcription of PA3559 as a member of the *arn* operon allows for more efficient modification of lipid A. It is therefore likely that, as a non-pathway-specific enzyme, a non-functional PA3559 would not have a deleterious effect on the development of a tolerance phenotype in *P. aeruginosa*, but further investigation would be needed to confirm this hypothesis.

This work also inspires questions about the role of disinfectants in antibiotic resistance. The widespread use of disinfectants provides ample opportunity for bacterial pathogens to become exposed to sub-lethal concentrations of disinfectants in a variety of settings. Despite a decline in the clinical use of polymyxins in the 1980s due to increased awareness of their nephrotoxicity, in recent years, polymyxin use has risen to treat increasing numbers of multi-drug-resistant bacterial infections [33]. Polymyxin B and colistin have, in recent years, been considered ‘last resort’ antibiotics. As an increasing number of patients require ‘last resort’ treatments with few new treatment prospects in sight, it is more important than ever to preserve the efficacy of these drugs. In carrying out further investigations, it would be important to demonstrate any potential increases in MIC of various clinical polymyxins following exposure to sub-inhibitory concentrations of disinfectants; in this case, it may be less relevant to use Opti-Free RepleniSH and may be of more use to test a sub-inhibitory concentration of a surface disinfectant. This would simulate the conditions under which a polymyxin-tolerant *P. aeruginosa* strain may be generated in a healthcare setting, where multi-drug-resistant infections are most common.

In the context of CL users, the potential increase in LPS modification by exposure to a sub-inhibitory concentration of Opti-Free RepleniSH poses potential risks. It is possible for CL users to inadvertently reduce the concentration of their multi-purpose disinfection solution through the use of water to rinse CL storage cases or by deliberately ‘topping up’ disinfection solutions instead of replacing with fresh solution each day. One study showed that 28% of CL users in the cohort reported never using fresh solution or occasionally reusing old solution; the same study showed that 52% of CL users in the cohort reported rinsing their lens cases with water [8]. These non-compliant behaviours increase the likelihood of inadvertently diluting the disinfection solution, thus exposing any contaminating microbes to a potentially sub-inhibitory concentration of the solution. When transferred to the eye via the CL, these contaminants would then be met by the innate immune response of the ocular surface, which includes a large number of CAMPs [34]. There are a wide variety of both α and β defensins produced at the ocular surface in the corneal epithelium, the conjunctival epithelium, the corneal stroma and the tear film, some of which are constitutively expressed and many of which have varied specificity against pathogenic organisms [34,36]. It is likely that CAMPs play a major role in the innate immunity of the ocular surface, and so a reduction in bacterial susceptibility to killing by CAMPs through LPS modification may increase the probability of a contaminating organism causing an infection in the cornea, once transferred to the ocular surface by the improperly disinfected CL.

## Conclusions

In this study, we have shown that *P. aeruginosa* has a propensity to alter its gene expression and translation to increase survival in the presence of disinfectants and that this phenotype can occur in both a laboratory (PAO1) and a clinical strain. Phenotypic tolerance is not easily detectable through modern culture- and sequence-based screenings but may play an important role in the development of disease. CAMPs are important both as antimicrobial compounds and as part of the human innate immune system, and so resistance and tolerance are mechanisms that should be investigated. Transcriptional and translational alterations that permit tolerance and resistance to CAMPs may also be implicated in resistance to last-resort antibiotics such as polymyxins.

## Supplementary material

10.1099/jmm.0.002073 Uncited Supplementary Material 1.

10.1099/jmm.0.002073 Uncited Supplementary Material 2.

## Abbreviations

Ara4N: 4-amino-4-deoxy-l-arabinose
CAMPs: cationic antimicrobial peptides
CL: contact lens
EDTA: ethylenediaminetetraacetic acid
IQR: interquartile range
LB: Luria broth
PBS: phosphate buffered saline
QACs: quaternary ammonium compounds
UDP: uridine diphosphate
