# Supplementary material for: Induction of lipid A modification genes in Pseudomonas aeruginosa cells tolerant to a commercially available contact lens disinfection solution
Source: J Med Microbiol. 2025 Oct 3;74(10):002073. doi: 10.1099/jmm.0.002073 (PMC12494483; doi:10.1099/jmm.0.002073)
Supplement: Uncited Supplementary Material 1. [file jmm-74-02073-s001.pdf]

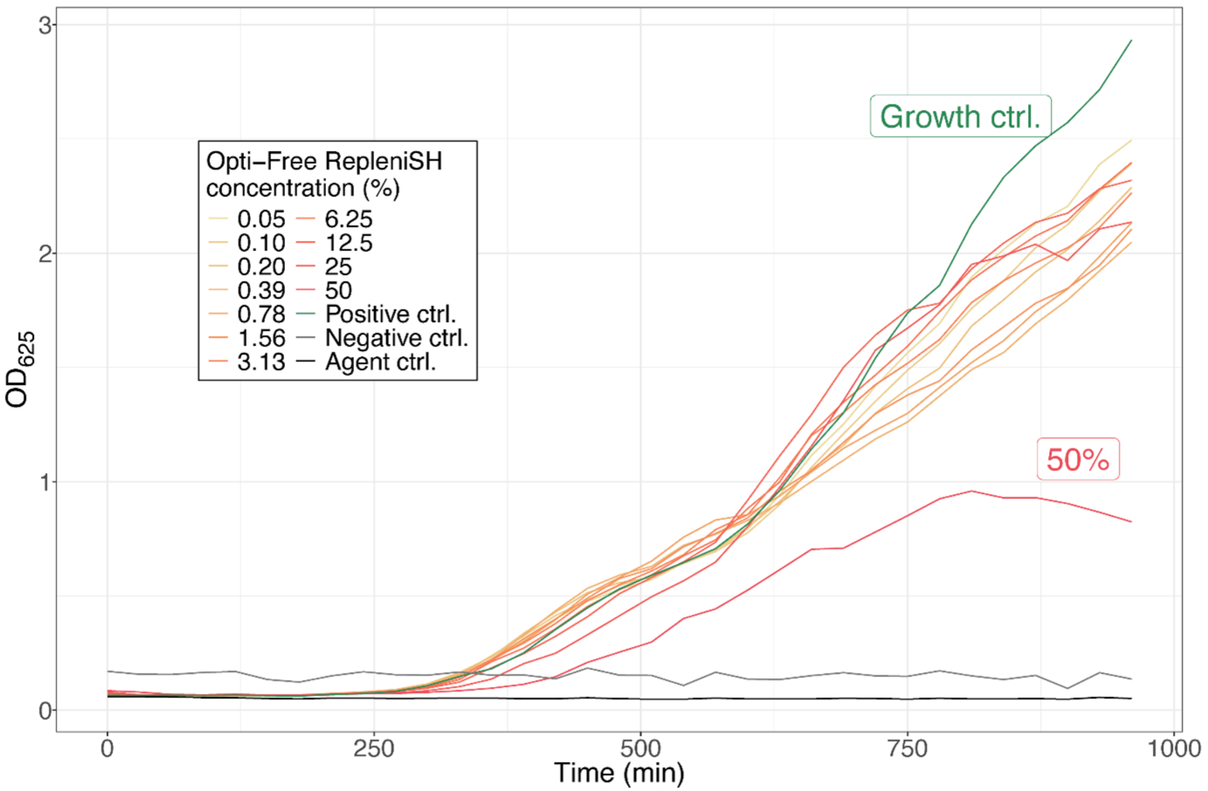

Figure S1: Growth curve data for determination of minimum inhibitory and sub-inhibitory concentrations of Opti-Free RepleniSH in LB broth against *P. aeruginosa* PAO1. Abbreviations: ctrl, control; min, minutes; OD_{625}, optical density at 625 nm.

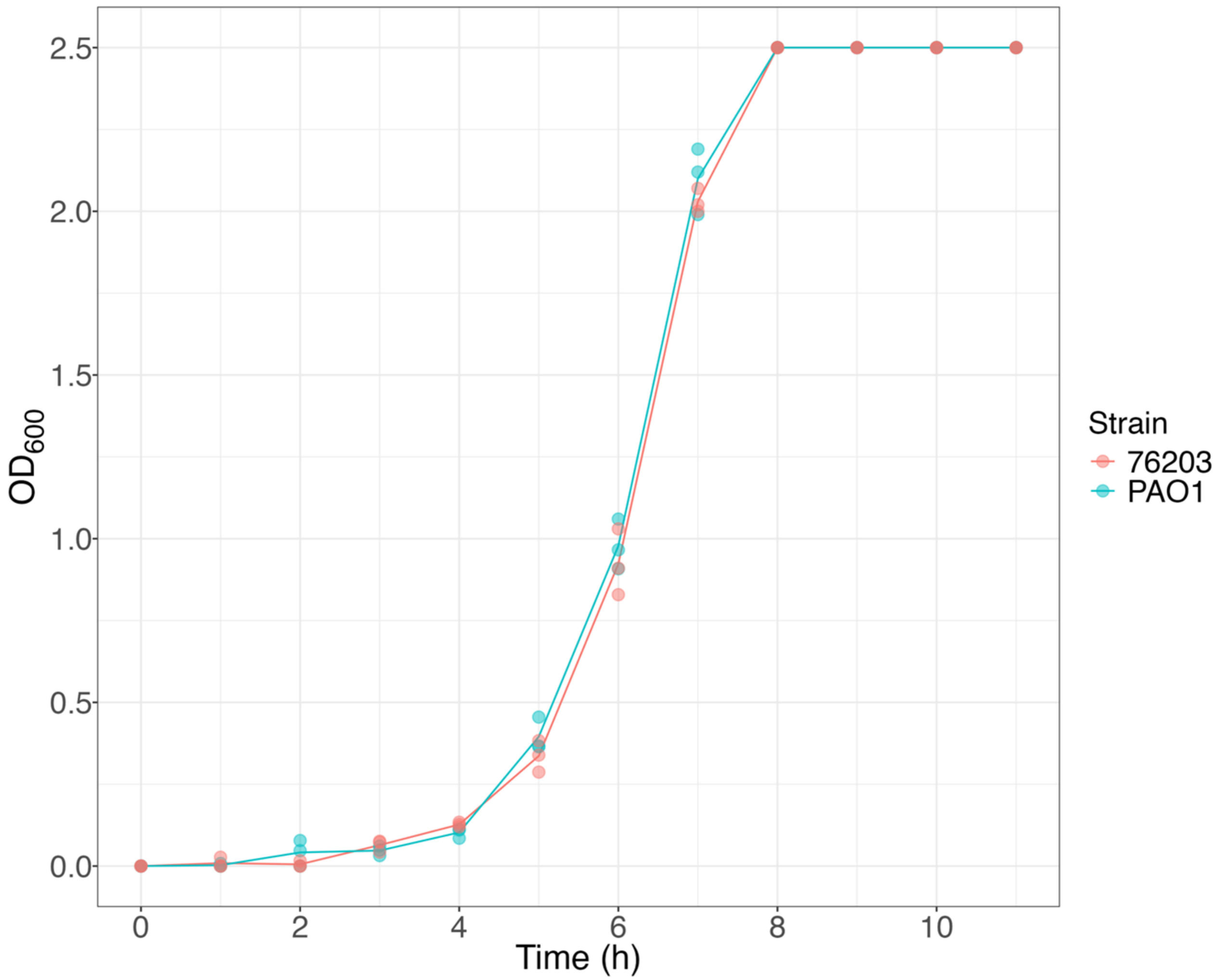

Figure S2: There is no growth defect in clinical isolate 76203 when grown in a sub-inhibitory concentration of Opti-Free RepleniSH. No significant difference shown by ANOVA (P = 0.436). Abbreviations: h, hours; OD_{600}, optical density at 600 nanometres.
